# Epigenetic silencing of tumor suppressor long non-coding RNA *BM742401* in chronic lymphocytic leukemia

**DOI:** 10.18632/oncotarget.12252

**Published:** 2016-09-26

**Authors:** Lu Qian Wang, Kwan Yeung Wong, Zhen Hai Li, Chor Sang Chim

**Affiliations:** ^1^ Department of Medicine, Queen Mary Hospital, The University of Hong Kong, Hong Kong

**Keywords:** BM742401, lncRNA, tumor suppressor, DNA methylation, chronic lymphocytic leukemia

## Abstract

*BM742401* is a tumor suppressor lncRNA downregulated in gastric cancer. As the promoter region and the entire transcript are embedded in a CpG island, we postulated that *BM742401* is a tumor suppressor lncRNA inactivated by DNA methylation in chronic lymphocytic leukemia (CLL). The promoter of *BM742401* was unmethylated in normal controls including three each of normal bone marrow, peripheral blood buffy coats, and CD19-sorted peripheral B-cells, but methylated in four (57.1%) CLL cell lines. Methylation of *BM742401* correlated inversely with expression. In the completely methylated WAC3CD5+ CLL cells, 5-Aza-2′-deoxycytidine treatment led to promoter demethylation and re-expression of *BM742401* transcript. Functionally, stable overexpression of *BM742401* resulted in inhibition of cellular proliferation and enhanced apoptosis through caspase-9-dependent intrinsic but not caspase-8-dependent extrinsic apoptosis pathway, suggesting a tumor suppressor role of *BM742401* in CLL. In primary CLL samples, methylation of *BM742401* was detected in 43/98 (43.9%) of patients. Moreover, among CLL patients with standard-risk cytogenetic aberrations, methylation of *BM742401* correlated with advanced Rai stage (≥ stage 2)(*P* = 0.002). Furthermore, *BM742401* methylation was associated with *miR-129-2* methylation (*P* = 0.05). *BM742401* is a tumor suppressor lncRNA frequently methylated in CLL. The mechanism of *BM742401* as a tumor suppressor warrants further studies.

## INTRODUCTION

DNA methylation refers to the addition of a methyl group (−CH_3_) to the carbon 5 position of the cytosine ring in a CpG dinucleotide, leading to the formation of 5-methylcytosine [1]. Global DNA hypomethylation but aberrant DNA hypermethylation of tumor suppressor genes (TSGs) is a hallmark of many human cancers [2]. To date, in hematological cancers, methylation-mediated silencing of multiple TSGs have been found involved in the dysregulation of multiple cellular pathways including *CDKN2A* and *CDKN2B* in the regulation of cell-cycle, *DAPK1* and *APAF1* in the regulation of apoptosis, and soluble *WNT* inhibitors involved in inhibition of WNT signaling [3–6]. Of note, methylation-mediated silencing of both protein-coding TSGs, including *DAPK1*, *ID4* and *SFRP1*, and tumor suppressor miRNAs, including *miR-9-3, miR-34b/c*, *miR-129-2*, *miR-203* and *miR-3151*, have been implicated in CLL leukemogenesis or prognosis [7–13].

Long non-coding RNA (lncRNA) is defined as a novel class of RNAs longer than 200 nucleotides with little or no protein-coding capacity. It can be further be categorized into five subgroups, including intergenic, intragenic, natural antisense transcripts, pseudogenes and unclassified transcripts, based on the location relative to annotated protein-coding genes (PCGs) [14, 15]. Moreover, lncRNAs are known to activate or inhibit gene expression involved in multiple biological processes through epigenetic, transcriptional or post-transcriptional mechanisms [16, 17].

In addition to PCGs and miRNAs, lncRNAs are found dysregulated in many human cancers, indicating their potential oncogenic or tumor suppressor role in carcinogenesis [15]. For example, HOX transcript antisense intergenic RNA (*HOTAIR*) is found overexpressed in breast cancer, in particular in patients with metastasis, suggestive of an oncogenic role in cancer metastasis [18]. Conversely, maternally expressed gene 3 (*MEG3*) is a tumor suppressor lncRNA downregulated in multiple human cancers, inhibiting cell proliferation through accumulation of TP53 protein by abrogating MDM2 function [19, 20]. Intriguingly, the promoter-associated CpG island of *MEG3* was found frequently hypermethylated in both solid and hematological malignancies, indicating that methylation-mediated silencing may serve as an alternative mechanism for inactivation of tumor suppressive lncRNAs [19].

In CLL, deletion of chromosome 13q14 is the most frequent chromosomal alteration detected in approximately 60% newly diagnosed patients, portending superior survivals [21]. In addition to *miR-15a/16-1* cluster, a lncRNA, *DLEU2* was found co-deleted in the minimally deleted region (DMR) of del(13q14), hence implicating a potential tumor suppressor role of *DLEU2*. Interestingly, downregulation of *DLEU2* in CLL has recently been ascribed to DNA methylation of its CpG islands [22]. Moreover, long intergenic non-coding RNA p21 (*lincRNA-p21*) and nuclear enriched abundant transcript 1 (*NEAT1*) were identified as transcriptional targets of TP53 and implicated in the TP53-depedent cell death after DNA damage in CLL [23]. However, apart from *DLEU2, lincRNA-21* and *NEAT1*, the role of lncRNAs in CLL remains unexplored.

Recently, *BM742401* has been found to be a tumor suppressor lncRNA inhibiting metastasis, and repression of *BM742401* was associated with inferior survivals in patients with gastric cancer [24]. Moreover, restoration of *BM742401* resulted in inhibition of metastasis and decreased secretion of extracellular MMP9, which enhances cell migration and invasion. As the promoter region and the entire transcript of *BM742401* are embedded in a CpG island, and the fact that methylation may serve as an alternative mechanism for silencing of tumor suppressor lncRNAs; herein, we postulated *BM742401* is a tumor suppressor lncRNA inactivated by DNA methylation in CLL.

## RESULTS

### Methylation of *BM742401*

#### Normal controls

**Figure 1:**
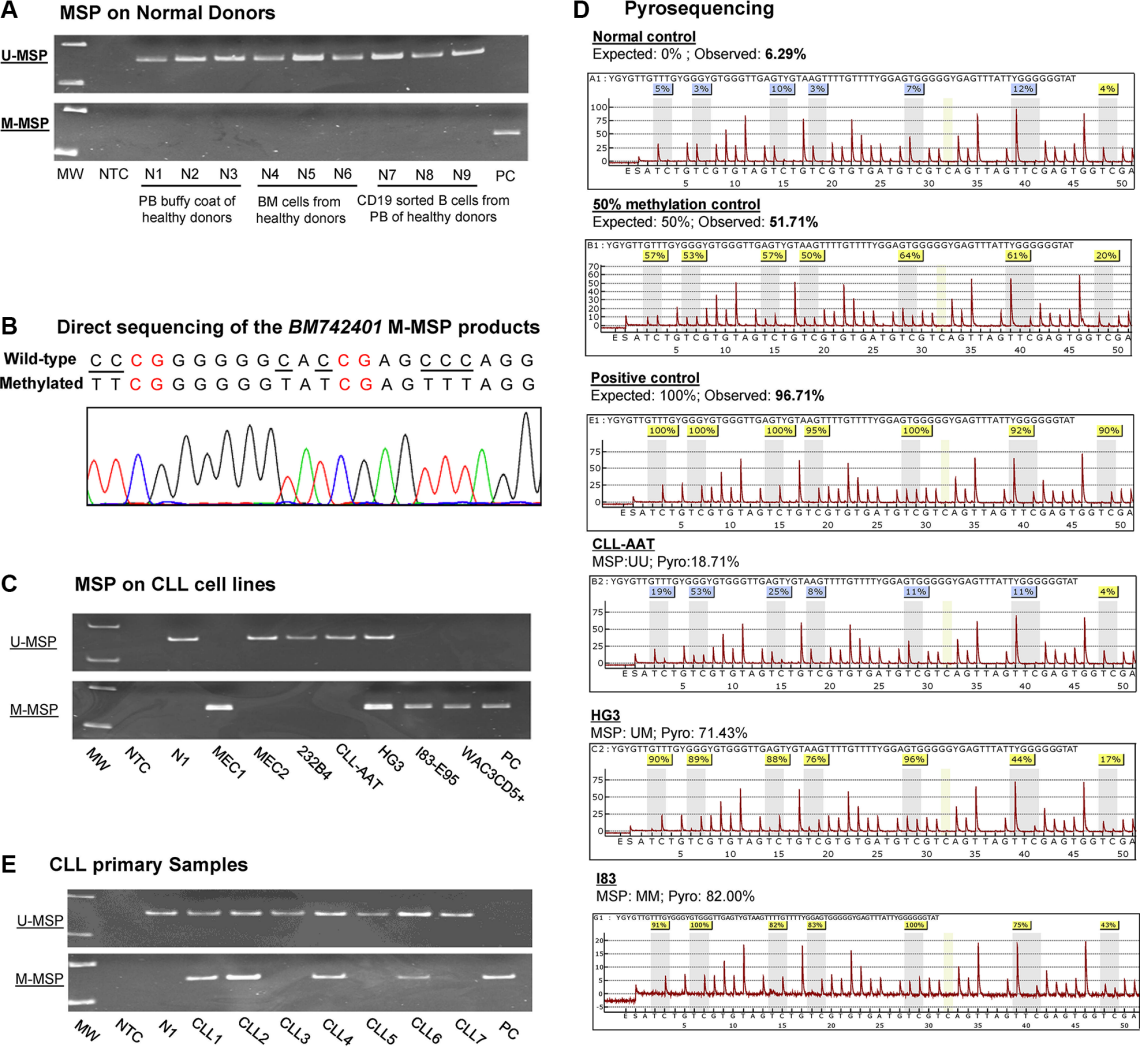
Methylation of *BM742401* (**A**) Methylation-specific PCR (MSP) showed that *BM742401* was completely unmethylated in normal controls. (**B**) Sequencing analysis of the M-MSP product from a methylated positive control showed the expected conversion of unmethylated cytosine to uracil (turned into thymidine after PCR) while leaving methylated cytosine unchanged, indicating the complete bisulfite conversion and specificity of MSP. The unmethylated cytosine was visualized by underlined text while the methylated cytosines highlighted in red. (**C**) MSP showed the methylation status of *BM742401* in a panel of CLL cell lines. (**D**) Pyrograms of quantitative bisulfite pyrosequencing showed the methylation intensity on a stretch of 7 neighboring CpG dinucleotides of *BM742401* in normal control, 50% methylation control, positive control with methylated DNA and CLL cell lines with defined MSP methylation status, complete methylation (MM), partial methylation (MU) and unmethylation (UU). (**E**) MSP showed *BM742401* methylation in primary CLL samples.

As illustrated in the schematic diagram showing the putative promoter region of *BM742401* (Supplementary Figure S1), methylation of *BM742401* was studied by MSP and pyrosequencing by primers designed upstream to the transcription start site. The promoter of *BM742401* was completely unmethylated in all of the nine normal controls (N1 to N9), but completely methylated in the positive control DNA (Figure 1A). Direct sequencing analysis of the M-MSP products amplified from the positive control showed expected conversion of unmethylated cytosine, but not methylated cytosine, into thymidine, indicating complete bisulfite conversion and specificity of the MSP (Figure 1B).

### CLL cell lines

In CLL cell lines, M-/U-MSP analysis showed that the promoter of *BM742401* was completely methylated (MM) in MEC1, I83-E95 and WAC3CD5+, partially methylated (MU) in HG3, and completely unmethylated (UU) in MEC2, 232B4, and CLL-AAT (Figure 1C). Moreover, the MSP methylation statuses MM, MU, and UU were confirmed by quantitative bisulfite pyrosequencing (Figure 1D). Together, these data suggested that methylation of *BM742401* was absent in normal controls but detected in four (57.1%) CLL cell lines, and hence tumor-specific.

### Primary CLL samples at diagnosis

By MSP, methylation of *BM742401* was detected in 43/98 (43.9%) of diagnostic CLL marrow samples (Figure 1E). Methylation of *BM742401* is found significantly associated with older age and higher diagnostic lymphocyte count (Supplementary Table S1). The mean age of patients with and without methylation of *BM742401* were 69 and 64 years old respectively (*P* = 0.03; Supplementary Table S1). The mean diagnostic lymphocyte count of patients with and without methylation of *BM742401* were 29 × 10^9^/L and 64 × 10^9^/L respectively (*P* = 0.04; Supplementary Table S1). However, there was no association between the methylation of *BM742401* and diagnostic hemoglobin level, platelet count, Rai stage, gender, death or high-risk karyotypes (Supplementary Table S1). The median OS of CLL patients with and without methylation of *BM742401* were 97 and 94 months respectively (*P* = 0.85; Supplementary Table S1). Moreover, among 44 low-risk CLL patients with del(13q14), normal karyotype or other karyotypic changes, methylation of *BM742401* significantly correlated with advanced Rai stage (≥ stage 2) (*P* = 0.002), but not survival. In addition, in 51 CLL patients with concomitant methylation study for both *BM742401* and *miR-129-2*, methylation of *BM742401* was significantly associated with that of *miR-129-2* (*P* = 0.05).

### Methylation and expression of *BM742401* in CLL cells

#### In CLL cell lines

**Figure 2:**
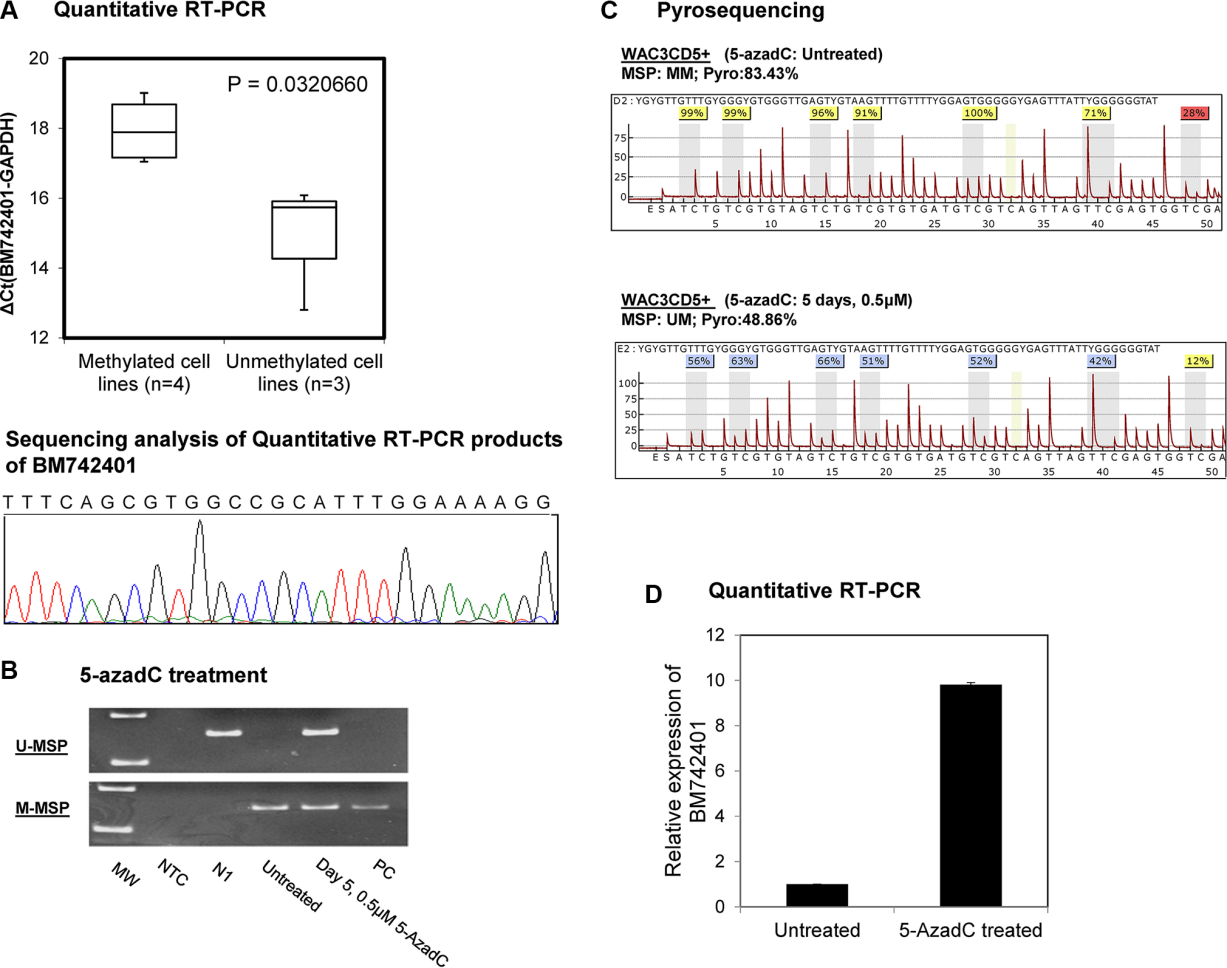
Methylation and expression of *BM742401* in CLL cells (**A**) Quantitative SYBR Green RT-PCR showed inverse correlation between *BM742401* methylation and expression in CLL cell lines. Sequencing analysis of the SYBR Green real-time RT-PCR products for the detection of *BM742401*. Treatment of CLL cells completely methylated for *BM742401* with 5-AzadC, a hypomethylating agent, (**B**) methylation-specific PCR and (**C**) quantitative bisulfite pyrosequencing showed *BM742401* was demethylated, and (**D**) quantitative RT-qPCR revealed *BM742401* was re-expressed.

CLL cell lines methylated for *BM742401* were significantly associated with lower expression of *BM742401*, as evidenced by RT-qPCR showing a higher ΔCt (Ct *BM742401*– Ct *GAPDH*), than those CLL cell lines completely unmethylated for *BM742401* (*P* = 0.032) (Figure 2A).

### 5-AzadC treatment of WAC3CD5+ cells

In WAC3CD5+ cells, which were completely methylated for *BM742401*, hypomethylation treatment with 5-AzadC led to the demethylation of *BM742401* promoter, as evidenced by the emergence of the U-MSP signal (Figure 2B) and decrease of the mean methylation percentage along the promoter CpG dinucleotides (Figure 2C) on day 5. Simultaneously, upon 5-AzadC treatment, expression of *BM742401* was increased in WAC3CD5+ cells (Figure 2D).

### Potential interplay between *BM742401* with neighboring *GATA6*

While lncRNA may regulate the expression of its neighboring gene [25], whether methylation-mediated silencing of *BM742401* is linked to its neighboring protein-coding gene *GATA6* was studied in CLL cell lines by qRT-PCR. Results showed that *GATA6* had a trend of lower expression in *BM742401*-methylated cell lines (MEC1, I83-E95 and WAC3CD5, HG3) than *BM742401*-unmethylated cell lines (MEC2, 232B4, and CLL-AAT) (*P* = 0.14) (Supplementary Figure S2), as indicated by a higher ΔCt (Ct *GATA6* − Ct *GAPDH*) in *BM742401*-methylated than *BM742401*-unmethylated CLL cell lines.

### Function of *BM742401* in CLL cells

**Figure 3:**
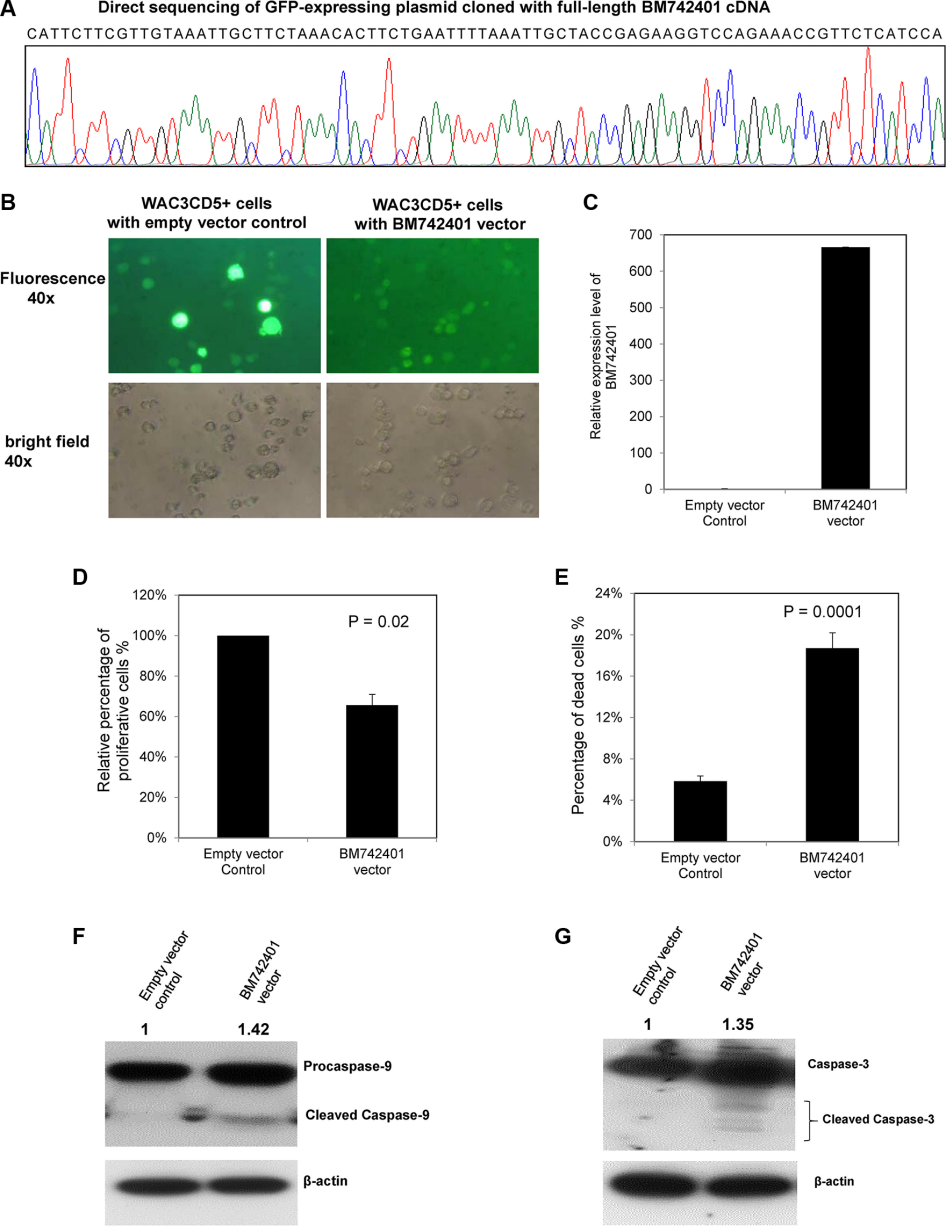
Function of *BM742401* in CLL cells Stable overexpression of *BM742401* in WAC3CD5+ CLL cells was performed by lentiviral infection of a GFP-expressing plasmid cloned with full-length *BM742401* cDNA. WAC3CD5+ CLL cells stably transduced with GFP-expressing empty plasmid were used as control. (**A**) Sequence analysis of GFP-expressing plasmid cloned with full-length *BM742401* cDNA. (**B**) Representative fluorescence (upper, 40× magnification) and bright field images (lower, 40× magnification) of WAC3CD5+ CLL cells stably transfected with empty vector or *BM742401* are shown. (**C**) Quantitative RT-qPCR for *BM742401* expression was performed. ΔCt, Ct *BM742401*-Ct *GAPDH*. GAPDH was chosen as reference using the 2^−ΔΔC^^T^ method. (**D**) Relative cell proliferation of CLL cells upon over-expression of *BM742401* was measured by MTT assay. (**E**) Percentage of dead cells was measured by Trypan blue exclusion assay. Data of MTT and Trypan blue assay shown were mean ± S.D. obtained from experiments in triplicate. Western blot analysis of (**F**) Caspase 9 and (**G**) Caspase 3 after *BM742401* overexpression. Total protein was extracted the same time after lentiviral transfection and membranes were probed with antibodies to anti-Caspase 3, -Caspase 9 and anti-actin. Signal from anti-actin antibody was used as the endogenous normalizer. The numbers above the bands indicated the relative protein expression measured by densitometric analysis.

As *BM742401* was frequently methylated in CLL cells, with repressed expression, we postulated that it might act as a tumor suppressive lncRNA. The tumor suppressor function of *BM742401* was studied by stable overexpression of *BM742401* in WAC3CD5+ CLL cells completely methylated for *BM742401* via lentiviral transduction (Figure 3B). By RT-qPCR, *BM742401* was confirmed to be overexpressed in WAC3CD5+ cells, as compared with WAC3CD5+ cells stably transduced with empty vector (Figure 3C). Moreover, over-expression of *BM742401* resulted in 34% reduction of cellular proliferation by MTT assay (*P* = 0.02, Figure 3D and Supplementary Figure S3A) and 13% increase of dead cells by Trypan blue exclusion assay (*P* = 0.0001, Figure 3E), as well as 11% increase of apoptotic cells indicated by cells in sub-G1 phase using propidium iodide (PI) staining (*P* = 0.005, Supplementary Figure S4A and S4B), indicating that *BM742401* is a tumor suppressor in CLL cells. Furthermore, restoration of *BM742401* led to increase of the activated, cleaved Caspase 9 and cleaved Caspase 3 (Figure 3F and 3G), but not cleaved Caspase 8 (p43/p41) (data not shown). Moreover, treatment with Z-LEHD-FMK, a caspase 9 inhibitor, rescued the percentage of dead cells by 9% in *BM742401*-overexpressing cells (*P* = 0.012, Supplementary Figure S3), but not in cells with empty vector, confirming the tumor suppressive role of *BM742401* is mediated by the intrinsic, Caspase-9 dependent apoptotic pathway.

To further confirm the tumor suppressor function of *BM742401*, the expression of *BM742401* in 232B4 cells, which were completely unmethylated for *BM742401*, was knockdowned by transfection of *BM742401*-specific 2′-O-Methyl phosphorothioate antisense oligonucleotides (2′OMe-PS ASOs). Upon transfection of *BM742401*-specific ASO1 and ASO2, the expression of *BM742401* was reduced by 56% and 66% respectively (Supplementary Figure S5A). Moreover, knockdown by *BM742401*-specific ASO1 led to 19.9% increase of cell proliferation by MTT assay (*P* = 0.04) (Supplementary Figure S5B) and 6% decrease of dead cells by Trypan blue exclusion assay (*P* = 0.03) (Supplementary Figure S5C), as compared with non-targeting control ASO. Furthermore, *BM742401*-specific ASO2-mediated depletion of *BM742401* led to 8% decrease of dead cells by Trypan blue exclusion assay (*P* = 0.03) compared with non-targeting control ASO (Supplementary Figure S5C).

## DISCUSSION

Several observations were made in this study. First, this is the first study demonstrating tumor-specific methylation of *BM742401* in CLL as shown by frequent methylation in CLL cell lines and primary CLL samples but not normal controls including normal CD19-sorted peripheral B-cells. Moreover, *BM742401* expression in CLL cell lines inversely correlated with methylation status. Furthermore, hypomethylating treatment in completely methylated CLL cells led to demethylation and re-expression of *BM742401*. Hence, methylation of *BM742401* promoter is associated with reversible silencing in CLL.

In addition to its frequent methylation in cell lines, *BM742401* was frequently methylated in primary CLL samples at diagnosis. Interestingly, there was a significant association of *BM742401* methylation with higher lymphocyte counts. Despite the lack of association between *BM742401* methylation and survival in CLL patients with low-risk cytogenetic aberrations, *BM742401* methylation correlated with the advanced Rai stage (≥ stage 2), which is a poor prognostic factor for CLL. Given the limited number of samples in our cohort, the prognostic impact of *BM742401* methylation in CLL warrants further studies. In light of the frequent methylation of *BM742401* methylation in primary CLL cells, *BM742401* methylation is important in CLL leukemogenesis. This contrasted with the frequent methylation of *miR-124-1* in myeloma cell lines but not primary myeloma plasma cells, hence excluding its role in pathogenesis of clinical myeloma [26].

Secondly, to gain insight into its biological function, *BM742401* was stably re-expressed in WAC3CD5+ cells with complete methylation of *BM742401*, which led to inhibition of cellular proliferation and enhanced cell apoptosis, indicating a tumor suppressive role in CLL. Caspases, a family of aspartate-specific cysteine proteases, are crucial mediators of apoptosis [27]. Cellular apoptosis may be activated by extrinsic/death receptor pathway or intrinsic/mitochondrial pathway [27]. In the extrinsic pathway, apoptosis is triggered upon activation of Caspase 8 upon ligation of membrane death receptors, such as members of the tumor necrosis factor (TNF) superfamily or Fas [28]. On the other hand, intrinsic apoptosis is triggered by the activation of Caspase 9 upon mitochondrial release of cytochrome C, resulting in the formation of apoptosome comprising Apaf-1, Caspase 9 and cytochrome C in the cytoplasm [29]. Once apoptosis is triggered, whether by extrinsic or intrinsic mechanism, both Caspase 8 and Caspase 9 leads to cleavage and hence activation of the effector Caspase 3 to initiate activation of the caspase cascade [30]. Herein, overexpression of *BM742401* resulted in the activation of Caspase 9 and Caspase 3, but not Caspase 8, showing that the tumor suppressive function of *BM742401* in CLL was mediated by activation of intrinsic apoptosis pathway.

Thirdly, *BM742401* gene exists in an antisense orientation to a neighboring protein-coding gene *GATA6*. Moreover, in CLL cell lines, methylation-mediated silencing of *BM742401* was linked to a lower expression of its neighboring protein-coding gene, *GATA6*. *GATA6* is implicated in human cancers as TSGs or oncogenes. In particular, loss-of-function *GATA6* in human astrocytes may lead to the acceleration of tumorigenesis, suggesting its tumor suppressor role in astrocytoma [31]. Conversely, *GATA6* overexpression could promote pancreatic carcinogenesis as an oncogene by activating the canonical Wnt signaling through antagonizing the expression of DKK1 at mRNA and protein level [32]. Indeed, recent studies showed that lncRNAs could regulate the expression of neighboring genes *in cis* or *trans* [25, 33]. For instance, *ANRIL*, an antisense transcript overlapping the *INK4b/ARF/INK4a* tumor suppressor locus, has been shown to repress its cognate transcript *INK4b in cis* through recruitment of polycomb repressive complex 1, and hence *INK4b/ARF/INK4a* repression [34]. The potential of molecular interaction between *BM742401* and its neighboring *GATA6* warrants further study.

Last but not least, an interesting observation was that methylation of *BM742401*, localized to chromosome 18q11.2 was associated with that of *miR-129-2* at chromosome 11q11.2 in CLL patients. Our previous study showed that *miR-129-2*, which targets sex determining region Y-box 4 (*SOX4*) and cyclin-dependent kinase 6 (*CDK6*), was frequently methylated in CLL at diagnosis [11, 35, 36]. Therefore, concomitant frequent methylation of both *BM742401* and *miR-129-2* might collaborate to enhance cell proliferation and survival, and hence CLL leukemogenesis. Intriguingly, recent reports have suggested that lncRNAs could modulate their regulatory roles through potential interaction with miRNAs [37]. Therefore, a potential role of *miR-129-2* in *BM742401*-associated tumor suppression in CLL warrants further studies.

Taken together, this study was first report of *BM742401* as a tumor suppressor lncRNA frequently methylated in CLL. Given the tumor suppressive role of *BM742401* in CLL, the exact mechanism of tumor suppressive function of *BM742401* warrant further studies.

## MATERIALS AND METHODS

### Patient samples

Bone marrow samples were obtained from 98 Chinese CLL patients diagnosed according to the standard morphologic and immunophenotyping criteria as described [10, 38]. Patient details and demographic data have been reported in our previous study and were listed in Table 1 [13]. The median overall survival (OS) of this cohort was 89 months. The median OS of those with advanced and limited Rai stage were 57 and 111 months respectively (*P* = 0.006). Moreover, the median OS for those with and without high/intermediate-risk karyotype were 43 months and 111 months respectively (*P* = 0.04). Of these, methylation of *miR-129-2* has been reported in 51 patients previously [11]. This study was approved by the Institutional Review Board of Queen Mary Hospital and samples were collected with written informed consent and in accordance with the Declaration of Helsinki.

**Table 1:** Demographic data of the 98 CLL patients at diagnosis

Characteristic	Value
**Gender**	
*Male*	67 (68.4%)
*Female*	31 (31.6%)
**Age**	
*Median*	67
*Range*	37–91
**Rai stage‡**	
*Limited Stage* (*stage 0/I/II*)	56 (62.2%)
*Advanced Stage* (*stage III/IV*)	34 (37.8%)
**Lymphocyte count**	
*Median*	16 × 10^9^/L
*Range*	10–540 × 10^9^/L
High-risk cytogenetics*	
*del(17p)*	3 (4.8%)
*del(11q)*	2 (3.2%)
*trisomy 12*	11 (17.7%)
Low-risk cytogenetics*	
*del(13)*	19 (30.6%)
*normal karyotype*	20 (32.4%)
*Other karyotype abnormalities*	7 (11.3%)

‡ from 90 patients with clinical data;

* from 62 patients with cytogenetic data.

### Cell lines and culture

The human CLL cell lines MEC1 and CLL-AAT were purchased from Deutsche Sammlung von Mikroorganismen und Zellkulturen Deutsche GmbH (DSMZ) (Braunschweig, Germany) and American Type Culture Collection (ATCC) (Manassas, USA) respectively. MEC2 [39] I83-E95 [40] and WAC3CD5+ [40] were kindly provided by Dr John C. Byrd, Department of Medicine, Ohio State University. HG3 and 232B4 were established and kindly provided by Prof. Anders Rosén, Department of Clinical & Experimental Medicine, Linköping University [41, 42]. Cell cultures were maintained in 90% RPMI 1640 + 10% fetal bovine serum, supplemented with 50 U/ml penicillin and 50 μg/ml streptomycin (Invitrogen, Carlsbad, CA, USA) in a humidified atmosphere of 5% CO_2_ at 37^°^C.

### Methylation-specific polymerase chain reaction (MSP)

DNA was extracted from 98 diagnostic marrow samples, seven CLL cell lines and nine healthy normal controls [buffy coats of peripheral blood (*N* = 3), bone marrow (*N* = 3), and CD19-sorted peripheral blood B-cells (*N* = 3)] by the QIAamp DNA Blood Mini Kit (QIAGEN, Germany). Bisulfite treatment of DNA was performed to convert unmethylated cytosine to uracil (but unaffecting methylated cytosine) using the EpiTect Bisulfite Kit kit (QIAGEN, Hilden, Germany). Each bisulfite-treated sample was amplified using primer sets specific to methylated DNA (methylated-MSP, M-MSP) and unmethylated DNA (unmethylated-MSP, U-MSP) respectively. Details of primers and conditions for M-MSP and U-MSP of *BM742401* were given in Table 2.

**Table 2:** Primer sequences and reaction condition

Gene	Forward primer (5′ to 3′)	Reverse primer (5′ to 3′)	Tm/cycles/MgCl_2_	References
**(I) Methylation-specific PCR (MSP)**				
*BM742401*				
U-MSP	TGTGTTGTTTAGGTAGATAAT GAGAGTTGT	CCAAATCAAACATTCT ATAACCTCCA	60°C/35 × /2 mM	
M-MSP	CGTTTAGGTAGATAATGAGA GTCGC	AAATCAAACGTTCTAT AACCTCCG	62°C/35 × /1.5 mM	
**(II) Reverse transcription-polymerase chain reaction (RT-PCR)**				
*BM742401*	TTGCTACCGAGAAGGTCCAG	GACATCCTTGTAGAAA AGAACCAA	60°C/40×	[24]
*GAPDH*	ACCACAGTCCATGCCATCACT	TCCACCACCCTGTTG CTGTA	60°C/40×	[44]
**(III) Cloning PCR**				
*BM742401*	GCTCTAGACATTCTTCGTTG TAAATTGCTTCTAA	GCTCTAGACGGAATTC AAAAGGAGCAATCAC TTATTTTTCC	56°C/40 × /2 mM	

Abbreviations: M-MSP, MSP for the methylated allele; U-MSP, MSP for the unmethylated allele; Tm, annealing temperature.

### Quantitative bisulfite pyrosequencing

Methylation-unbiased primers were designed by PSQ Assay Design software (Biotage) and used to amplify the promoter region of *BM742401*, encompassing the amplicon of MSP, in the bisulfite-treated DNA. Forward primer: 5′-AGGGGAGGAGAGAAAAGAG-3′; biotinylated reverse primer: 5′-AACTATACACTACCAAC TCCT-3′; condition: 2 mM/61°C/50X. A stretch of DNA with 7 consecutive CpG dinucleotides was pyrosequenced using sequencing primer: 5′-GTTTAGGTAGATAA TGAGAGT-3′.

### 5-Aza-2′-deoxycytidine (5-AzadC) treatment

WAC3CD5+ cells at log-phase were cultured with 0.5 μM of 5-AzadC (Sigma-Aldrich, St. Louis, MO, USA) for 5 days in a six-well plate at a density of 1 × 10^6^ cells/ml. Fresh 5-AzadC was replaced every 24 hours. Cells treated with 5-AzadC on day 0 and day 5 were harvested for further analysis.

### Plasmid construction and lentiviral transduction of *BM742401*

The full-length cDNA of *BM742401* was amplified and cloned into the XbaI and EcoRI sites of a pCDH-CMV-MCS-EF1-copGFP lentivector (System Biosciences; LV500A-1) (Table 2). The insert sequence was confirmed by direct sequencing (Figure 3A). According to the manufacturer's instructions, the *BM742401*-containing construct and pPACK packaging plasmid mix were then co-transfected into 293TN cells, followed by collection of supernatant at 48 hours after transfection. The supernatant (200 μl) was added into WAC3CD5+ cells and cultured for 72 hours. GFP-positive WAC3CD5+ cells were selected by flow cytometry (BD FACSAria I Cell Sorter) and further cultured for two weeks. WAC3CD5+ cells transduced with GFP-expressing empty vector plasmid were used as negative control.

### Knockdown of *BM742401* by antisense oligonucleotides (ASO)

*BM742401*-specific 2′-O-Methyl phosphorothioate antisense oligonucleotides (2′ OMe-PS ASOs) were designed, chemically synthesized and purchased from Integrated DNA Technologies (Coralville, IA, USA). ASO1 (*BM742401*-2′OMe/PS-320): mG*mA*mA*mA*mA*G*A*A*C*C*A*A*A*A*G*mC*mA*mA*mG*mG; ASO2 (*BM742401*-2′OMe/PS-96): mG*mG*mA*mG*mG*C* A*A*A*G*T*G*G*A*A*mC*mG*mU*mA*mA; non- targeting ASO control: mG*mC*mG*mU*mA*T*T*A*T* A*G*C*C*G*A*mU*mU*mA*mA*mC. Knockdown of *BM742401* was performed in 232B4 cells, which were plated at a density of 1 × 10^6^/ml in a 12-well plate and transfected with *BM742401*-ASO1, *BM742401*-ASO2 or non-targeting control ASO at a final concentration of 10nM using Lipofectamine 2000 (Invitrogen), according to the manufacturer's instructions. Cells were cultured and analyzed at 48 hours after transfection. The efficiency of knockdown was determined by qRT-PCR of *BM742401*.

### Quantification of *BM742401*

Total RNA was extracted by mirVana miRNA Isolation Kit (Ambion, Austin, TX, USA) and reverse transcribed by QuantiTect Reverse Transcription Kit (QIAGEN, Valencia, CA). *BM742401* was quantified using SYBR Green Master Mix (ABI, Foster City, CA, USA), following the manufacturer's instructions. The 2^−ΔΔCT^ method was used to detect the expression of *BM742401* before and after *BM742401* over-expression (or 5-AzadC treatment) in WAC3CD5+ cells [43]. The ΔCT method is used for comparing the *BM742401* expression between methylated and unmethylated CLL cell lines. *GAPDH* was used as endogenous control for data analysis of *BM742401*. Primers and conditions used for detection of *BM742401* were summarized in Table 2.

### MTT assay, Trypan blue dye exclusion assay and cell cycle analyses

The tumor suppressor function of *BM742401* was studied by MTT and Trypan blue dye exclusion assay. In brief, cells from each stably transduced sample were seeded in a 96-well microtitre plate at 2 × 10^5^ cells/well in 100 μl of medium. After 72 hours, each well was added 10 μl of 5 mg/ml MTT reagent (Sigma-Aldrich) and incubated for four hours. Then each well was added with 100 μl dimethyl sulfoxide (DMSO), followed by the measurement of absorbance at 550 nm with reference to 650 nm. The percentage of dead cells was measured by Trypan blue dye exclusion assay under microscope. Five random microscopic fields were counted for each sample. Dead cells (%) = (total number of dead cells per microscopic field / total number of cells per microscopic field) × 100. Cell cycle analysis was conducted by propidium iodide (PI) staining. Briefly, cells were washed in phosphate buffered saline (PBS), fixed in cold 70% ethanol at 4°C overnight, washed twice in PBS, resuspended and then incubated in 50 μg/ml PI staining solution with 5 μg/ml RNase A at 4°C for at least 2 hours, followed by the analyze of flow cytometry (Beckman Coulter Cytomics FC 500). Each sample was done in duplicate. MTT assay and Trypan blue dye exclusion assay were performed in triplicates of each sample. Data were plotted by mean ± standard deviation and compared by Student's *t-test*.

### Treatment with Caspase 9 inhibitor

WAC3CD5+ cells transduced with GFP-expressing empty vector or *BM742401* vector were treated with 10 μM Z-LEHD-FMK (Caspase 9 inhibitor, R&D system) for 3 days in a 12-well plate at a density of 1 × 10^6^ cells/ml respectively. After 72 hours, empty or *BM742401* vector cells with or without treatment were harvested for Trypan Blue exclusive assay.

### Western blotting

Seventy-two hours after equal number of cells (2 × 10^6^) from stably transduced samples were seeded in a six-well plate, total proteins (20 μg) were isolated at for Western Blot analysis, as described [10]. The primary antibodies incubated at 4°C overnight were anti-Caspase 3 (1:1000; Cell Signaling), -Caspase 8 (1:1000; Cell Signaling), -Caspase 9 (1:1000; Cell Signaling) and anti-actin (1:5000; Sigma-Aldrich, USA), followed by incubation with anti-rabbit or anti-mouse horseradish peroxidase conjugate secondary antibody at room temperature for 1 hour. ECL plus Western blotting detection reagent was used for the detection of protein signals with X-ray film (Amersham Biosciences, Buckinghamshire, UK). Protein bands were quantified using densitometry as measured by Quantity One 4.6.2 software (Bio-Rad); hence relative protein expression was expressed in comparison to corresponding control.

### Statistical analysis

In 98 primary CLL samples, the correlation between *BM742401* methylation with continuous (mean age, mean lymphocyte counts, diagnostic hemoglobin or platelet counts) and categorical variables (gender, Rai stage or high-risk karyotypes) were analyzed by student's *t-test* and chi-square test (or Fisher's exact test) respectively. OS is measured from the date of diagnosis to the date of last follow-up or death. Survival was plotted by the Kaplan-Meier method and compared by the log-rank test. OS of CLL patients with limited Rai stage (stage 0/I/II) was compared with those with advanced Rai stage (stage III/IV). Moreover, OS of CLL patients with high-risk karyotypes [del(17p), del(11q) or trisomy 12] was compared to those with standard-risk karyotypes [del(13q), normal karyotype or other karyotypic changes]. Association between methylation of *BM742401* and *miR-129-2* was studied by chi-square test. All *P* values were two-sided.

## SUPPLEMENTARY MATERIALS


