# Supplementary material for: Epigenetic silencing of tumor suppressor long non-coding RNA BM742401 in chronic lymphocytic leukemia
Source: Oncotarget. 2016 Sep 26;7(50):82400–10. doi: 10.18632/oncotarget.12252 (PMC5347700; doi:10.18632/oncotarget.12252)
Supplement: Supplementary file 1 [file oncotarget-07-82400-s001.pdf]

Epigenetic silencing of tumor suppressor long non-coding RNA *BM742401* in chronic lymphocytic leukemia

Supplementary Materials

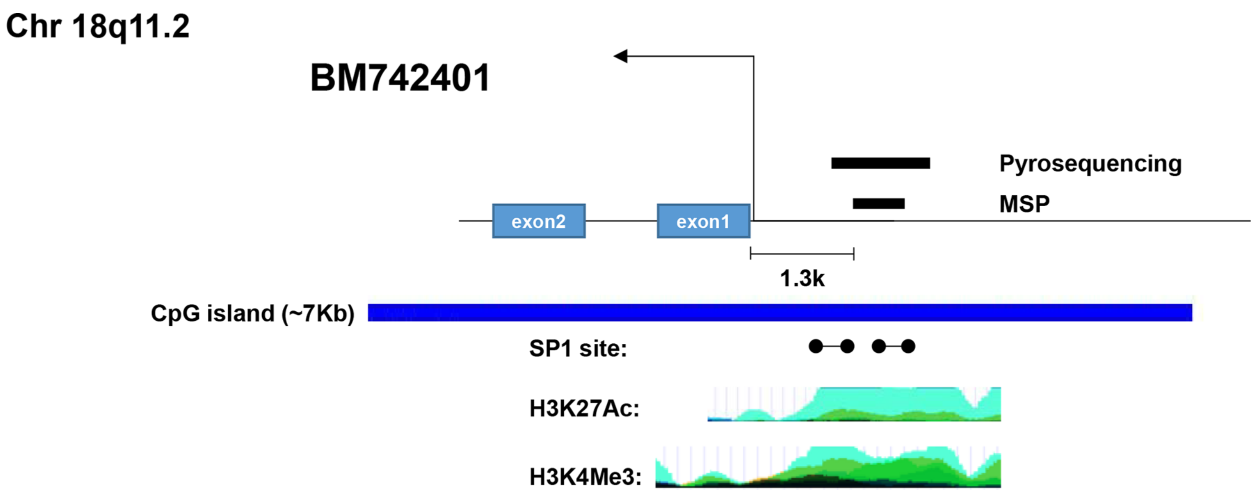

Supplementary Figure S1: Schematic diagram of lncRNA *BM742401*.

It showed that the whole transcript, including the putative promoter region, of *BM742401* is embedded in a CpG island of 7426 bp length. Based on data from UCSC Genome Browser, the putative promoter region of *BM742401* shows occupancy of active promoterassociated histone code, including H3K4Me3 and H3K27Ac [1, 2], and SP1 binding sites [3] near the regions (black bars) of MSP and pyrosequencing.

REFERENCES

1. Shlyueva D, Stampfel G, Stark A. Transcriptional enhancers: from properties to genome-wide predictions. Nat Rev Genet. 2014; 15:272–286.
2. Ernst JP, Kheradpour TS, Mikkelsen N, Shoresh LD, Ward CB, Epstein X, Zhang L, Wang R, Issner M. Coyne. Mapping and analysis of chromatin state dynamics in nine human cell types. Nature. 2011; 473:43–49.
3. Briggs, MR., JT, Kadonaga SP, Bell R. Tjian. Purification and biochemical characterization of the promoter-specific transcription factor, Sp1. Science. 1986; 234:47–52.

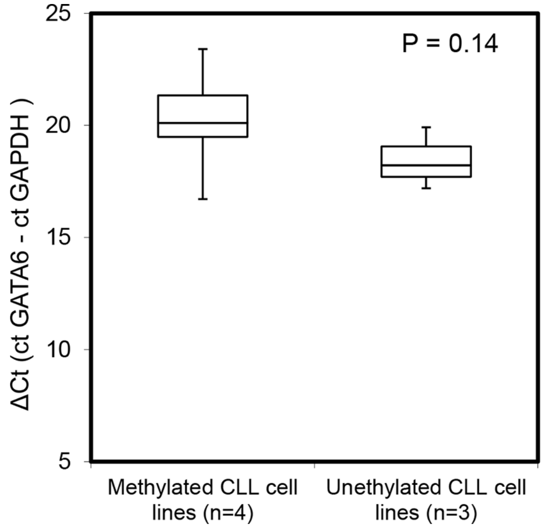

Supplementary Figure S2: GATA6 expression in BM742401-methylated and BM742401-unmethylated CLL cell lines. Quantitative SYBR Green RT-PCR showed a lower GATA6 expression in *BM742401*-methylated CLL cell lines than *BM742401*-unmethylated. ΔCt, Ct *GATA6-Ct GAPDH. GAPDH* was used as reference for data analysis by ΔCt method.

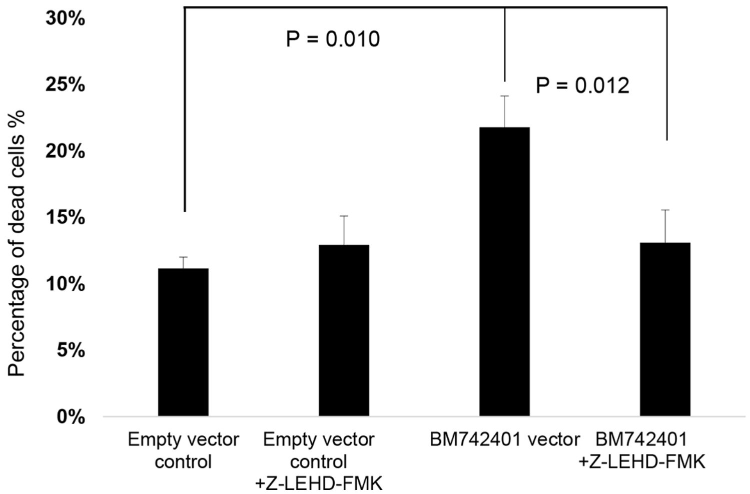

Supplementary Figure S3: Treatment with Caspase 9 inhibitor on WAC3CD5+ cells with or without BM742401 overexpression. WAC3CD5+ cells with GFP-expressing empty vector or *BM742401* vector were treated with 10 µM Z-LEHD-FMK, a Caspase 9 inhibitor, for 3 days. Percentage of dead cells was measured by Trypan blue exclusion assay. Column, mean percentage of dead cells from two independent experiments with triplicate in each. Error bars represent standard deviation.

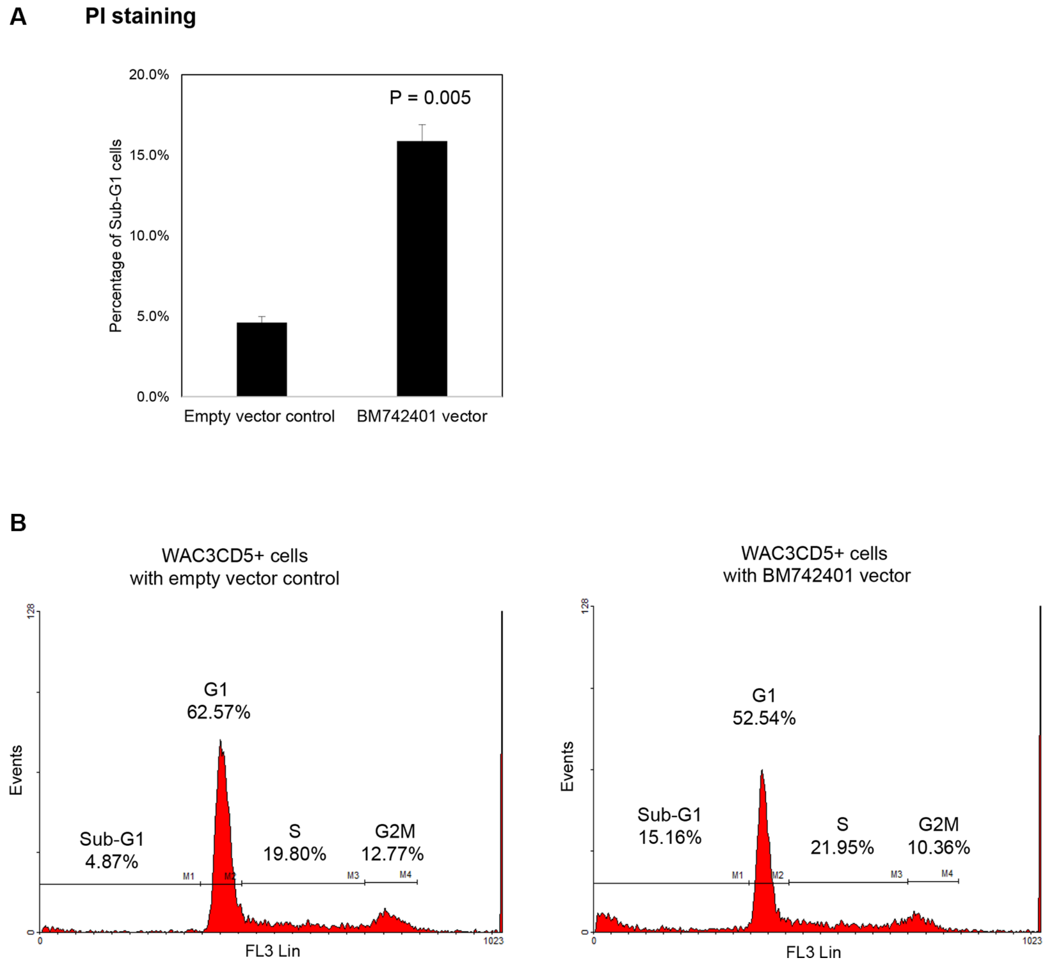

Supplementary Figure S4: Cell cycle analysis upon *BM742401*-overexpression in CLL cells. (**A**) Stable overexpression of *BM742401* in WAC3CD5+ cells was performed by lentiviral infection and percentage of apoptotic cells in sub-G1 phase measured by the propidium iodide staining was plotted. (**B**) Representative flow cytometric histogram of PI-stained WAC3CD5+ cells with empty vector control and *BM742401* vector in WAC3CD5+ cells.

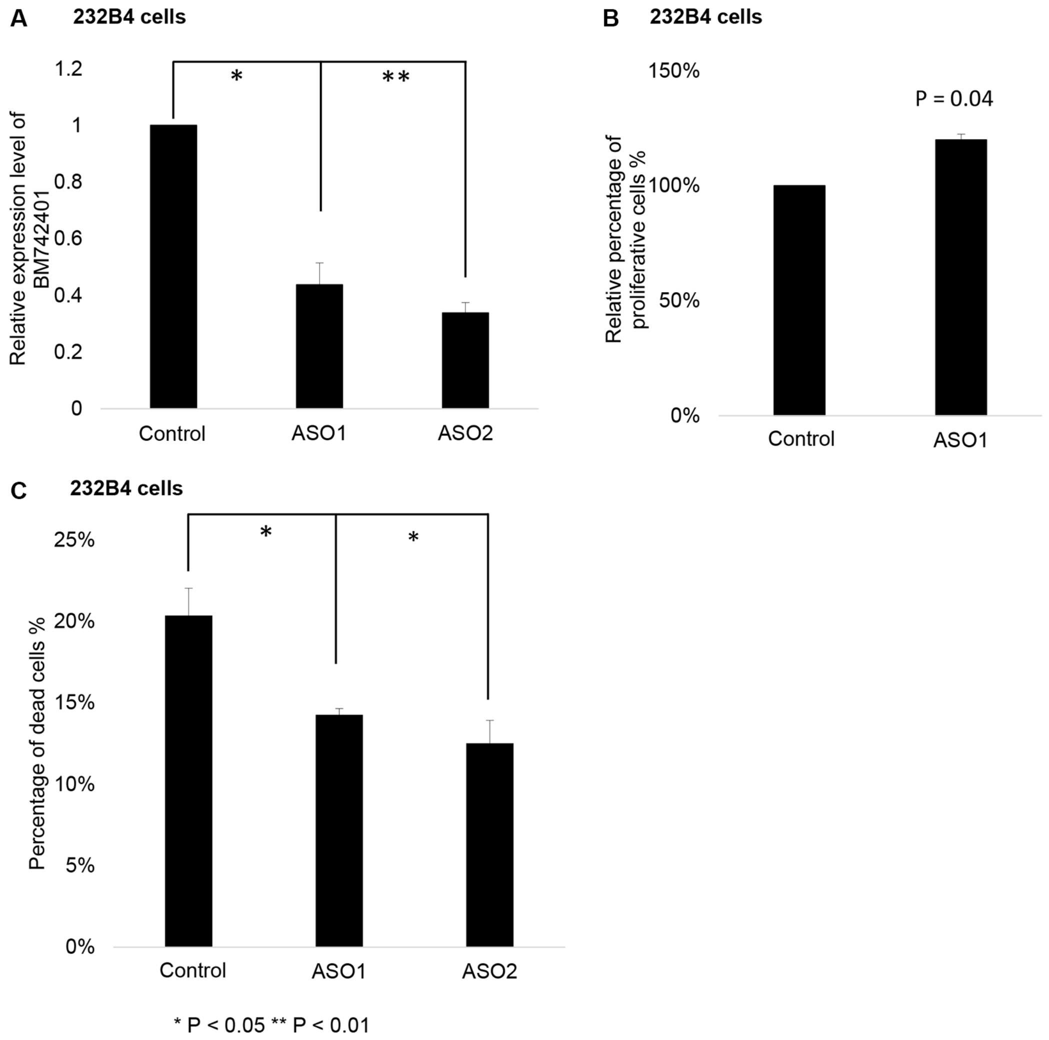

Supplementary Figure S5: Knockdown of *BM742401* by Antisense oligonucleotides. Knockdown of *BM742401* was performed by transfecting 232B4 cells, which expressed and completely unmethylated of *BM742401*, with *BM742401*-specific ASO1 (2′OMe/PS-320) or ASO2 (2′OMe/PS-96), as compared with non-targeting control ASO. Cells were harvested 48 hours after transfection. (**A**) qRT-PCR showed the expression of *BM742401* upon transfection with *BM742401*-ASO1/ASO2 or non-targeting control ASO, in 232B4 cells. *GAPDH* was used as reference for data analysis of *BM742401* expression by $2^{-\Delta\Delta CT}$ method. (**B**) Relative cell proliferation of CLL cells upon knockdown of *BM742401* using ASO1 was measured by MTT assay. (**C**) Percentage of dead cells upon knockdown of *BM742401* using ASO1 or ASO2 was measured by Trypan blue exclusion assay. Data of MTT and Trypan blue were mean ± S.D. from two transfection experiments with triplicate in each. *$P < 0.05$ **$P < 0.01$.

Supplementary Table S1: Correlation of methylation of *BM742401* with clinical parameters in CLL patients

	BM742401 methylated patients	*BM742401* unmethylated patients	*P* value
**Mean age (yrs)**	69 (43/98)	64 (55/98)	*P* = 0.03
**Diagnostic lymphocyte count (/L)**	29×10^9 (42/96)	64×10^9 (54/96)	*P* = 0.04
**Diagnostic hemoglobin (g/dL)**	10.81 (42/96)	11.53 (54/96)	*P* = 0.208
**Platelet counts (/L)**	175.38×10^9 (42/96)	226.11×10^9 (54/96)	*P* = 0.349
**Median overall survival (months)**	97 (43/98)	94 (55/98)	*P* = 0.85
**Gender**			
Male	27.6% (27/98)	40.8% (40/98)	*P* = 1.000
Female	13.3% (13/98)	18.3% (18/98)	
**Death**			
Death	19.4% (19/98)	23.5% (23/98)	*P* = 0.840
Not death	24.5% (24/98)	32.6% (32/98)	
**HR karyotype**			
*Low-risk cytogenetics**	33.9% (21/62)	40.3% (25/62)	*P* = 0.780
*High-risk cytogenetics**	12.9% (8/62)	12.9% (8/62)	
**Rai stage**			
Limited Stage (stage 0/I/II)	23.3% (21/90)	38.9% (35/90)	*P* = 0.126
Advanced Stage (stage III/IV)	21.1% (19/90)	16.7% (15/90)	
